# Role of chromosome stability and telomere length in the production of viable cell lines for somatic cell nuclear transfer

**DOI:** 10.1186/1471-213X-6-41

**Published:** 2006-08-09

**Authors:** Gabriela F Mastromonaco, Steve D Perrault, Dean H Betts, W Allan King

**Affiliations:** 1 Department of Biomedical Sciences, Ontario Veterinary College, University of Guelph, Guelph, Ontario, N1G 2W1, Canada

## Abstract

**Background:**

Somatic cell nuclear transfer (SCNT) provides an appealing alternative for the preservation of genetic material in non-domestic and endangered species. An important prerequisite for successful SCNT is the availability of good quality donor cells, as normal embryo development is dependent upon proper reprogramming of the donor genome so that embryonic genes can be appropriately expressed. The characteristics of donor cell lines and their ability to produce embryos by SCNT were evaluated by testing the effects of tissue sample collection (DART biopsy, PUNCH biopsy, post-mortem EAR sample) and culture initiation (explant, collagenase digestion) techniques.

**Results:**

Differences in initial sample size based on sample collection technique had an effect on the amount of time necessary for achieving primary confluence and the number of population doublings (PDL) produced. Thus, DART and PUNCH biopsies resulted in cultures with decreased lifespans (<30 PDL) accompanied by senescence-like morphology and decreased normal chromosome content (<40% normal cells at 20 PDL) compared to the long-lived (>50 PDL) and chromosomally stable (>70% normal cells at 20 PDL) cultures produced by post-mortem EAR samples. Chromosome stability was influenced by sample collection technique and was dependent upon the culture's initial telomere length and its rate of shortening over cell passages. Following SCNT, short-lived cultures resulted in significantly lower blastocyst development (≤ 0.9%) compared to highly proliferative cultures (11.8%). Chromosome stability and sample collection technique were significant factors in determining blastocyst development outcome.

**Conclusion:**

These data demonstrate the influence of culture establishment techniques on cell culture characteristics, including the viability, longevity and normality of cells. The identification of a quantifiable marker associated with SCNT embryo developmental potential, chromosome stability, provides a means by which cell culture conditions can be monitored and improved.

## Background

In recent years, the development of novel embryo production technologies including somatic cell nuclear transfer (SCNT) has created the potential for the restoration and propagation of valuable genetic lines, and the preservation of endangered species/breeds. Although numerous procedural parameters remain to be streamlined to increase the success of this technique for the production of live and normal offspring, one of the important prerequisites of SCNT is the availability of healthy donor cells, which need to be reprogrammed to support proper embryo and fetal development after nuclear transplantation.

The last decade has seen a dramatic increase in the cryopreservation of biomaterials. Genome resource banks have been established for the collection and storage of somatic/gonadal tissues, cells, gametes and embryos from genetically valuable individuals and lineages. It is intended that these banks will be instrumental in the genetic management of captive and wild populations. Wildt [1] stated that genome resource banks will not only facilitate the dispersal of genetic material among populations, but will provide insurance against the sudden loss of diversity or a population. However, lack of standardized techniques for sample collection and storage has resulted in an abundance of poor-quality banked material. Thus, a better understanding of the effects of tissue collection and culture establishment techniques on cell viability is required. To ensure that cells are of the utmost quality for possible use as donors for SCNT, a need exists for the identification of quantifiable markers with a direct relationship to developmental potential.

It is well known that most somatic cells grown in vitro have a limited proliferative lifespan [2], at which point they enter a phase of growth arrest termed replicative senescence. This irreversible state is distinguishable from the quiescence achieved by serum-starvation or density-dependent growth inhibition [3] that is necessary to synchronize donor cells in the G_o _phase for SCNT. Senescent cells remain metabolically active, arrested at the G1/S boundary and lacking the ability to divide [reviewed by [4]]. A senescent phenotype, whereby the cells change in form and function, has been described. The characteristics include alterations in morphology (increased cell surface and volume, flattening of the cell), function (over-expression of cytokines) and gene expression patterns, resistance to apoptosis and shortened telomere length [reviewed by [5]]. In vitro lifespan is affected by genetic and environmental factors, such as donor age, physiological state, culture conditions and seeding density [5-7].

Prolonged serial cultivation of cells in vitro results in the accumulation of numerous aberrations. Genomic instability and telomere shortening have both been correlated with increased time in culture [8-10], and shown to be prevalent at replicative senescence. A dramatic increase in chromosomally abnormal cells in late passage cultures has been demonstrated by various investigators [10-12]. Benn [10] observed that the chromosome content of cell lines progressed from approximately 30% abnormal to greater than 60% abnormal cells in later passages. Similarly, Strelchenko [11] and Giraldo et al. [12] reported 81% and 98% abnormal cells in late passage cultures, respectively. Both structural and numerical abnormalities have been documented, including end-to-end fusions, translocations, aneuploidy and polyploidy.

An important role in genomic stability is carried out by the telomeres [13]. Telomeres are known to maintain chromosome structure and function by preventing end-to-end fusions and degradation of the chromosome ends [14]. As telomeres shorten with each cell cycle, the "sticky" ends are prone to fusions and translocations leading to structural chromosome alterations and subsequent chromosomal instability [15]. Numerical chromosome alterations, on the other hand, are thought to result from the missegregation of chromosomes due to the loss of p53 and Rb, regulators of the mitotic spindle apparatus [15]. Both chromosome abnormalities and critically short telomeres are thought to be able to signal the irreversible damaged DNA response that causes the cells to enter the phase of growth arrest leading to senescence [4,16].

In vitro production of embryos, particularly by SCNT, is associated with compromised pre- and post-implantation development. Cytogenetic studies on embryos indicate that chromosome abnormalities are associated with poor embryo quality and increased embryonic and fetal wastage [17]. Chromosome abnormalities are thought to be a major cause of embryonic loss during early development [18,19]. Among in vitro fertilized (IVF) and SCNT embryos, a higher percentage of chromosomally abnormal blastomeres were observed in slower developing embryos, embryos with lower cell numbers and poorer grade embryos [17,20,21]. Studies have correlated the occurrence of chromosome abnormalities in SCNT embryos with the frequency of abnormalities in the donor cell line [22,12]. Not surprisingly, donor cell lines with a higher percentage of abnormal cells generated a higher rate of chromosomally abnormal blastomeres in the SCNT embryos.

In order to improve SCNT success, careful attention must be given to the establishment of viable, normal cell lines since good quality embryos depend upon the chromosomal integrity of the donor cell. Due to the numerous environmental factors influencing the long-term growth of a cell line, standardization of techniques for tissue collection, processing and culture must be implemented to ensure that banked cultures are a viable source of genetic material. Proper cell banking techniques are of critical importance in non-domestic species, specifically endangered wildlife, where access to the animals is minimal and, in some cases, precludes the collection of good quality tissue samples.

The objectives of this study were to investigate the effects of tissue sample collection and cell culture initiation techniques on cell culture dynamics and their effects on the developmental potential of cloned embryos following SCNT. Our aim was to identify cell culture parameters that would serve as predictors of the developmental potential of a cell line in the initial stages of in vitro culture, for selecting and banking samples that would provide the best outcome as donor cells for SCNT. In addition, to determine the feasibility of cell banking in a non-domestic species requiring specialized handling techniques the experiments were performed on an endangered cattle species, the gaur (*Bos gaurus*), using domestic cattle (*Bos taurus*) as controls.

## Results

**Figure 1:**
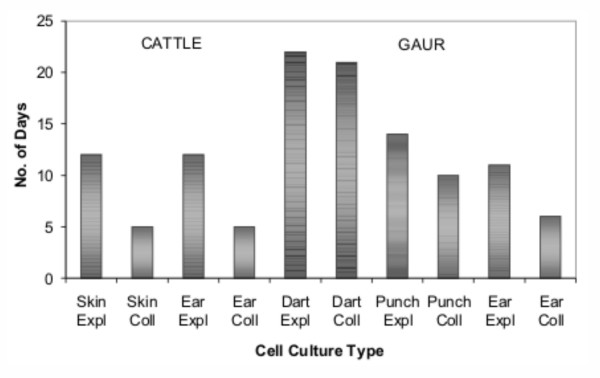
**Length of time to reach primary confluence for cattle and gaur cell cultures**. The numbers of days necessary for the dissociated cells to become confluent monolayers are shown for the sample collection (DART, PUNCH, EAR/SKIN) and cell dissociation (EXPL, COLL) treatment groups. Time to reach confluency varied according to the sample collection and cell dissociation techniques used.

Culture characteristics were examined for DART, PUNCH and EAR/SKIN samples established using EXPL and COLL techniques. The time to primary confluence varied according to the sample collection technique used (Figure 1). Small initial samples, as in DART, required >20 days to achieve confluence compared to larger samples, as in EAR, which required only 5–6 days (COLL) and 11–12 days (EXPL). In all cases, EXPL treatments required more time than COLL to achieve confluence.

**Figure 2:**
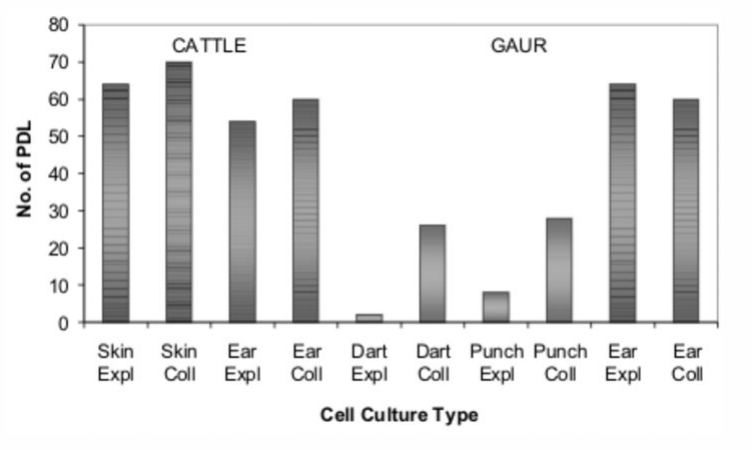
**Replicative lifespan of cattle and gaur cell cultures**. The numbers of population doublings to reach senescence are shown for the sample collection (DART, PUNCH, EAR/SKIN) and cell dissociation (EXPL, COLL) treatment groups. Differences in the number of population doublings prior to senescence were observed between the sample collection techniques and only between cell dissociation techniques when short-lived cultures were produced.

Cultures were passaged routinely until they reached senescence and differences were observed in the number of PDL to senescence among the treatments (Figure 2). The cattle controls (SKIN and EAR) and gaur EAR produced long-lived cultures with >50 PDL. In these cases, no significant effect of cell dissociation technique was evident after long-term culture. Both DART and PUNCH produced short-lived cultures (<10 PDL, EXPL; <30 PDL, COLL). In these cases, cell dissociation technique did have an effect, with COLL cultures undergoing more PDL than EXPL cultures.

**Figure 3:**
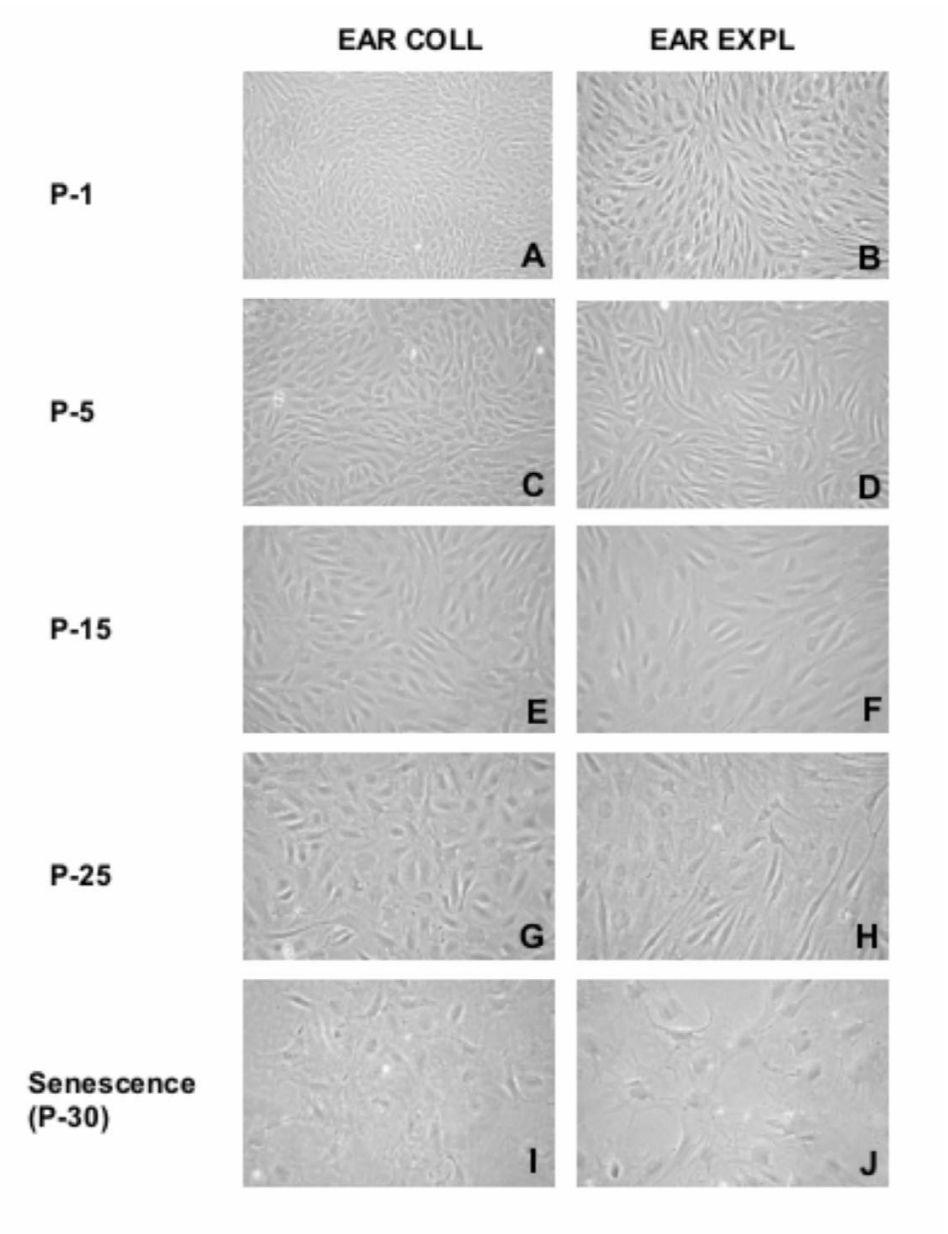
**Morphology of gaur cell cultures during serial cultivation in vitro**. Phase contrast micrographs of gaur EAR fibroblast cultures from early and late passages demonstrate the changes in cell morphology that occurred with increased passages. Note the morphology (enlarged and flattened cells) characteristic of senescent cultures in (I) and (J). No major effects of cell dissociation techniques were observed in these long-lived cell cultures.

**Figure 4:**
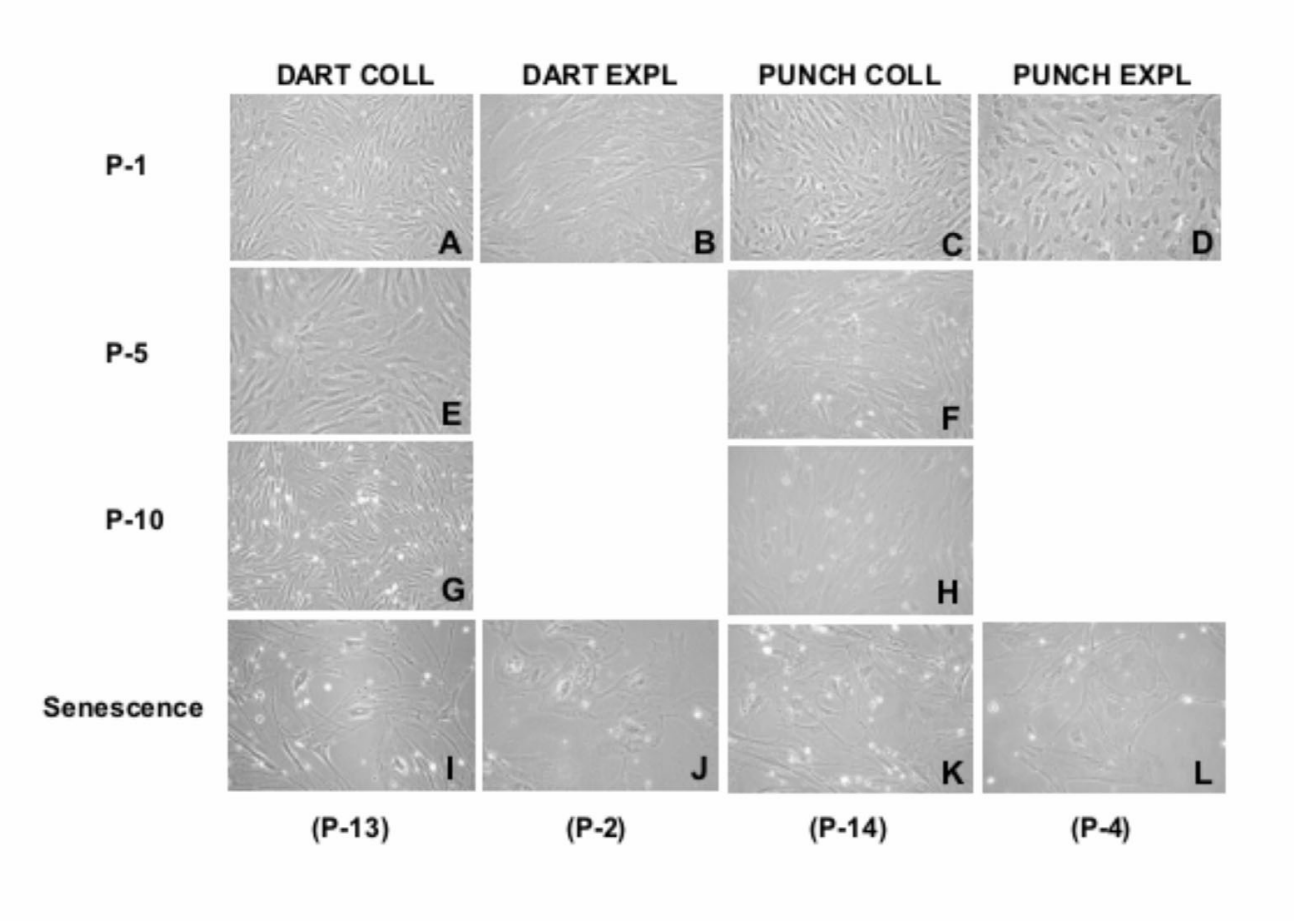
**Morphology of gaur cell cultures during serial cultivation in vitro**. Phase contrast micrographs of gaur DART and PUNCH fibroblast cultures from early and later passages demonstrate changes in cell morphology. Senescent morphology (enlarged and flattened cells) was observed within the first few passages as shown in (I) to (L). Effects of cell dissociation techniques were apparent in these short-lived cell cultures.

To examine phenotypic changes during serial cultivation, culture morphology was assessed. Long-lived cultures (gaur EAR, Figure 3; cattle SKIN and EAR not shown) exhibited a typical trend from the tightly compact spindle-shaped cells in early culture (Figure 3A–D) to a gradual enlarging of cells until the spread out, star-shaped phenotype of senescence was observed (Figure 3I–J). In contrast, within the first few passages, short-lived cultures (DART and PUNCH, Figure 4) displayed the phenotypic changes (enlarging and spreading) characteristic of the late passage cells of long-lived cultures approaching senescence.

**Figure 5:**
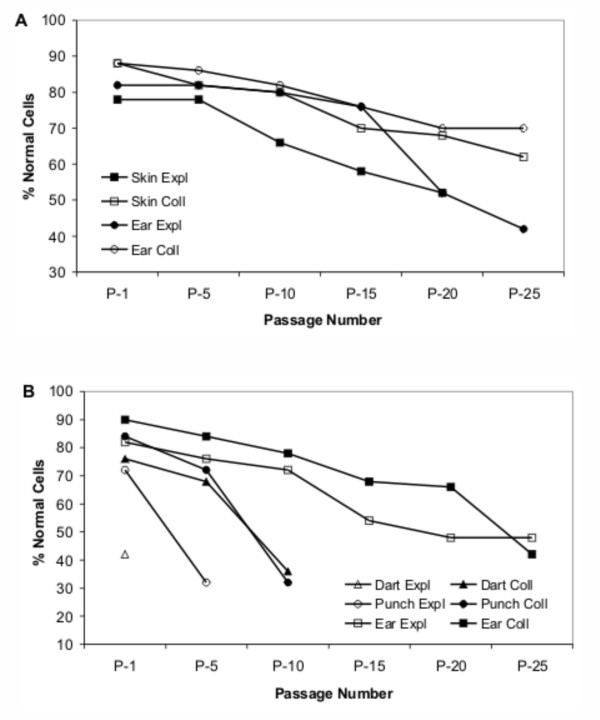
**Chromosome content of cell cultures during serial cultivation in vitro**. Percentages of chromosomally normal cells (% Normal Cells) were assessed for (A) cattle and (B) gaur cell cultures. A decrease in chromosomally normal cells occurred in all cultures with increased passages. Differences in the initial percentage and the rate of decline of chromosomally normal cells were observed in the different treatment groups. Effects of sample collection techniques were clearly evident in the gaur cell cultures.

To examine changes in genomic stability during serial cultivation, chromosome content of the cultures was assessed. Analysis of metaphase spreads at P-30 was not successful due to the low numbers of actively dividing cells in the late passage cultures. A decrease in the percentage of chromosomally normal cells was evident in all cultures during serial cultivation (Figure 5A–B). Abnormalities included primarily aneuploidy and polyploidy. The initial percent normal cells, final percent normal cells, and rate of change (slope) varied between cultures, indicating differences due to both sample collection and cell dissociation techniques. Long-lived cultures (cattle SKIN/EAR, Figure 5A; gaur EAR, Figure 5B) showed a gradual decrease (slope range: -0.75 to -2.00, Table 1) in chromosomally normal cells from 82–90% normal down to 42–70% normal by passage 25 (50 PDL). Short-lived cultures (DART and PUNCH, Figure 5B) initially consisted of a lower percentage of chromosomally normal cells and dropped dramatically (slope range: -4.44 to -10.00, Table 1) within the first few passages to <40% normal cells. In general, EXPL cultures resulted in a lower percentage of normal cells compared to COLL cultures. The DART EXPL cell line failed to establish and was not analyzed beyond P-1, which already consisted of only 42% normal cells.

**Table 1:** Rate of change (slope) of chromosome content and relative telomere length following serial cultivation in vitro.

Animal	Cell Culture	CC	RTL
CATTLE	SKIN EXPL	-1.51	-0.06
	SKIN COLL	-1.07	-0.04
	EAR EXPL	-1.75	-0.03
	EAR COLL	-0.82	-0.04

GAUR	DART EXPL	-	-
	DART COLL	-4.52	-0.04
	PUNCH EXPL	-10.00	-0.17
	PUNCH COLL	-5.87	-0.05
	EAR EXPL	-1.60	-0.02
	EAR COLL	-1.79	-0.11

**Figure 6:**
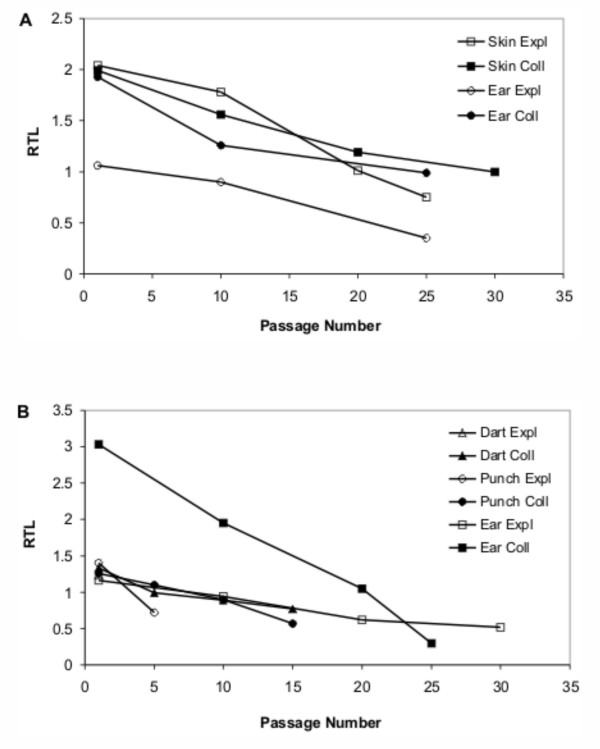
**Relative telomere lengths of cell cultures during serial cultivation in vitro**. Relative telomere lengths (RTL) were assessed for (A) cattle and (B) gaur cell cultures. A decrease in RTL occurred in all cultures with increased passages. Effects of sample collection and cell dissociation techniques were not clearly evident.

Slopes were obtained following linear regression analysis of the data in figures 5 and 6. Effects of sample collection and cell dissociation techniques on CC slopes, but not RTL slopes, were detected in the gaur cell cultures. CC = chromosome content; RTL = relative telomere length.

To examine the changes in telomere length during serial cultivation, relative telomere length of the cultures was assessed. A decrease in RTL was evident in all cultures during serial cultivation (Figure 6A–B). Definitive differences due to sample collection or cell dissociation techniques were not clearly evident, however some trends were observed. The initial and final RTLs varied between cattle and gaur. With one exception, RTLs of cattle cultures were greater than those of gaur cultures. More importantly, initial and final RTLs of long-lived cultures were approximately twice that of short-lived cultures, with the exception of cattle EAR EXPL and gaur EAR EXPL which were both lower than other long-lived cultures. In most cases, the rate of telomere shortening (slope) was similar (Figure 6A–B, Table 1). The DART EXPL cell line failed to establish and was not analyzed beyond the initial RTL at passage 1.

Chromosome stability was tested for dependence on the initial RTL, final RTL, and RTL rate of change to determine if a significant relationship exists. Furthermore, differences between the animals, sample collection techniques and cell dissociation techniques were tested. The chromosome content rate of change was not found to be dependent on any one telomere length factor alone, but was highly dependent on both the initial RTL of the culture (p < 0.05) and the RTL rate of change (p < 0.001) together. Inclusion of the animal, sample collection technique and cell dissociation technique class variables revealed that while cell dissociation technique was not significant, animal and sample collection technique were both significant (p < 0.05) factors.

**Figure 7:**
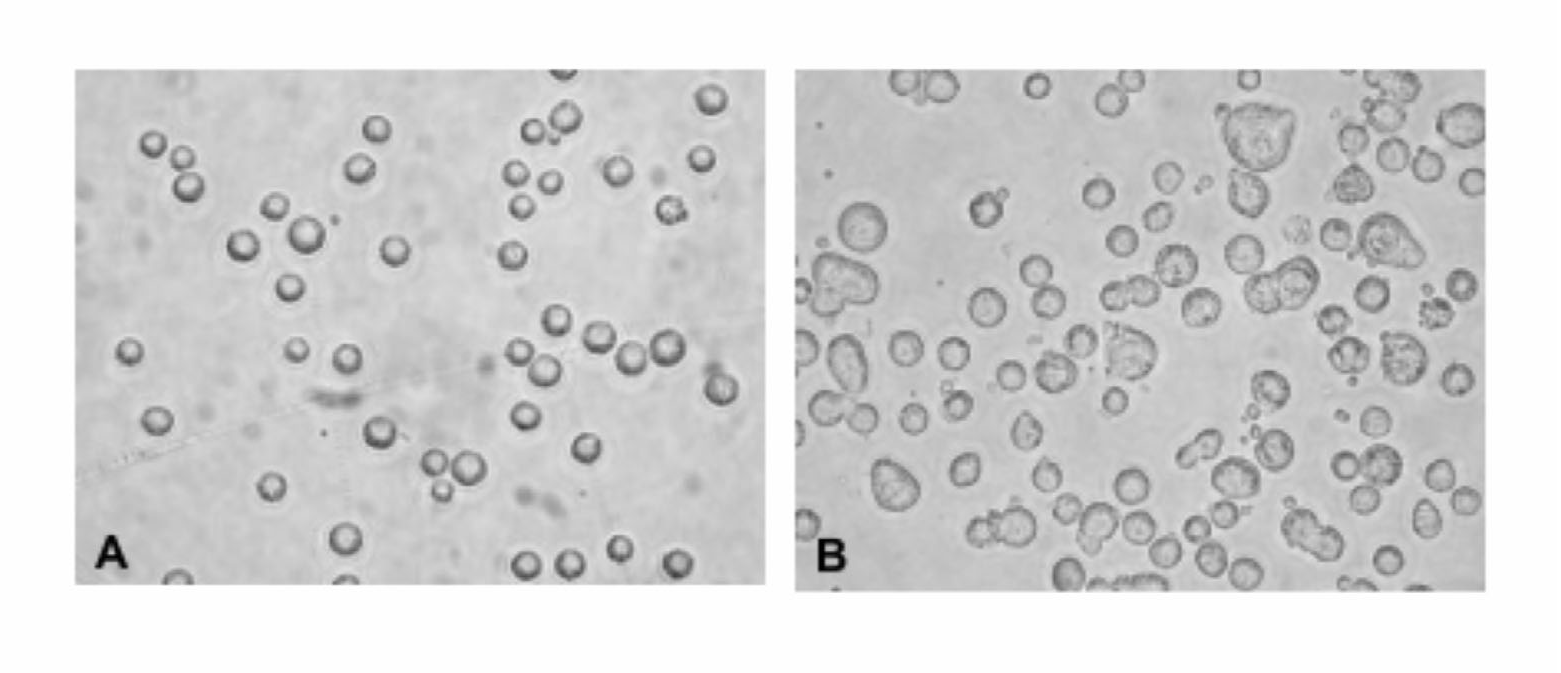
**Morphology of cattle EAR cell preparations for SCNT**. Phase contrast micrographs of (A) early passage (P-5) and (B) late passage (P-30) fibroblast cultures prepared for SCNT demonstrate changes that occur following prolonged growth in vitro. A marked increase in cells with enlarged size and abnormal morphology was observed in the late passage culture.

The effects of cell culture quality on the production of embryos by SCNT were examined (Table 2). Only COLL cultures were used as EXPL cultures from DART and PUNCH samples did not produce enough cells for the SCNT experiments. In the cattle controls, SKIN and EAR gave similar results, with approximately 35% blastocyst development on day 8 of embryo culture. When near-senescent cattle cells (SKIN and EAR, P-30) were used for SCNT, significantly (p < 0.05) lower development was observed, with <11% blastocyst development for both cultures. Interestingly, when cells were trypsinized and prepared for SCNT, morphological differences were evident between the early and late passage cells (Figure 7). Early passage cell preparations consisted of small, spherical, smooth-membraned cells, with a small percentage of the cells appearing abnormal (larger in size with non-spherical or ragged edges). In the late passage cell preparations, a higher proportion of cells displayed an abnormal appearance. Blastocyst rates for gaur EAR cells averaged 11.8%. Since the gaur embryos being produced were the result of interspecies SCNT, gaur embryo development was expected to be lower than cattle. However, a comparison among the gaur cell lines used for SCNT showed that the short-lived DART and PUNCH cultures produced significantly (p < 0.05) lower development and these cell preparations were similar in appearance to the late passage cell preparations (figure not shown).

**Table 2:** SCNT embryo development using collagenase digestion cultures.

Animal	Cell Culture	Total Embryos (N)	Day 8 Blastocyst (%)
CATTLE	SKIN COLL	117	36.1 ± 3.4^a^
	SKIN COLL P-30	121	10.7 ± 1.0^b^
	EAR COLL	94	34.7 ± 3.4^a^
	EAR COLL P-30	76	1.4 ± 1.4^b^

GAUR	DART COLL	74	0.8 ± 0.8^a^
	PUNCH COLL	90	0.9 ± 0.9^a^
	EAR COLL	154	11.8 ± 1.3^b^

A significant decrease in blastocyst development was observed in reconstructed embryos from short-lived and near-senescent cell lines. These data indicate that cell culture parameters influence embryo developmental competence following SCNT. N = total number of reconstructed embryos (successfully fused couplets) placed in culture. All cultures for SCNT were prepared at P-3 to P-5 unless otherwise stated. ^a, b ^Superscripts within columns denote a significant difference between treatments (p < 0.05).

Following the determination that chromosome stability was highly dependent on a combination of initial RTL and RTL rate of change, we examined the possibility that the blastocyst development rate of cell lines used for SCNT was dependent on the chromosome stability of the donor cell line. Indeed, chromosome content rate of change and sample collection technique were significant (p < 0.01) factors in determining blastocyst development outcome.

## Discussion

This study demonstrated that the methods of tissue sample collection and cell culture initiation influence the viability, longevity and normality of cells grown in vitro, and their subsequent potential for embryo development following SCNT. Furthermore, chromosome stability was identified as a quantifiable parameter on which developmental potential of SCNT embryos is highly dependent. The strong relationship between chromosome stability and somatic/embryonic cell viability makes this cell characteristic a valuable parameter for predicting SCNT success, and provides a means by which cell culture conditions can be monitored and improved.

### Effects on culture characteristics

Culture-specific factors exert significant effects on the proliferative lifespan of cell cultures, with seeding density being proportionately related to lifespan [6,7]. Similarly, in our study differences resulting from the sample collection techniques showed that the initial sample size, that is, the number of cells potentially available to initiate the culture (seeding density), had an effect on the amount of time necessary for achieving primary confluence and the number of PDL produced. Rapidly dividing, long-lived cultures were only obtained from skin and ear samples collected post-mortem. In the smaller samples from biopsies obtained by dart or manual punch establishment of the cell lines was also affected by the cell dissociation technique. The longer time to primary confluence and the significantly reduced lifespans of the explanted cell lines may be the result of a smaller number of cells migrating out of the explants compared to those released by enzyme digestion. Thus, these lower-density cultures may exhaust more of their replicative potential in the initial phase of culture. Although small biopsies are routinely used in humans to produce cultures of >40 PDL [23], the thickness and toughness of skin in large animals like cattle may be a factor for consideration when attempting to obtain the full thickness of the epidermal and dermal layers.

Although an age discrepancy existed between the cattle and gaur samples as slaughterhouse animals are usually less than 5 years old, whereas the gaur was 18 years old, it did not have an effect on the observed results. Gaur EAR samples obtained primary confluence within the same time frame as cattle EAR and, similarly, produced long-lived cell lines with 60 or more PDL. Cristofalo et al. [23] observed that although fetal and adult fibroblasts had different growth characteristics, no effect of donor age on replicative lifespan was evident. The data reported here were generated from samples obtained from one gaur individual due to the difficulty in obtaining both biopsy and post-mortem samples from this endangered species. However, similar outcomes were obtained from DART biopsies from 8 other gaur and from PUNCH biopsy and EAR from one other gaur (personal observations).

A relationship between the amount of time to primary confluence and the number of PDL was observed, with cultures being successfully established only from cell preparations that achieved confluence within the standard time-frame for that technique (explants: 10–12 days; digestion: 5–6 days). Cultures requiring >10 days to form a confluent monolayer following enzyme digestion were indicative of cell lines that underwent replicative senescence prematurely. This shows that monitoring the initial phase of culture establishment provides important information for determining the reproductive capabilities of a cell line and deciding whether to proceed with the culture. Bartels et al. [24] banked cell cultures from African lions (Panthera leo) whose mean time to confluence in 50 ml flasks following enzyme digestion was 18.6 ± 3.1 days. Although no information is available on the viability of these cultures, our data suggest that these slow-growing cultures will not produce healthy, highly proliferative cell lines.

Replicative senescence is accompanied by a change in the phenotype of the culture [5]. Alterations in morphology are described as an increase in cell surface and volume (i.e. size) and a flattening of the cell, both evident by light microscopy. Cell morphology was affected by the growth rate and lifespan of the culture and, therefore, influenced by the sample collection and cell dissociation techniques as described above. Long-lived cultures exhibited a gradual change in morphology with increased passage number as observed in skin and ear samples collected post-mortem. Major changes were only evident within the last 10 PDL. Cultures that failed to establish successfully displayed the senescence phenotype initially or within the first few PDL. This overt change in morphology suggests that visual observation of a culture's phenotype can be an effective indicator of the replicative potential of a cell line.

Senescent cells have been reportedly characterized by high levels of chromosome abnormalities [25]. Increased proportions of aneuploid, polyploid and dicentric chromosomes have been documented with increasing passage number [10-12,26,27]. In one study the percentage of aneuploid cells ranged from 61.5% to 100% in late passage cells [27]. Our results similarly demonstrate that chromosome abnormalities become increasingly prevalent in cells approaching the end of their replicative lifespan. Rapidly dividing, morphologically normal cells had a high percentage of chromosomally normal cells, in most cases >80% normal. After a prolonged time in culture, the number of chromosome abnormalities gradually increased, becoming more prevalent in late passage cells with up to 58% abnormal cells as the cultures approached senescence. However, in the poorly established cultures resulting from small initial samples a high percentage of chromosome abnormalities were observed initially or within 10 PDL. Consequently, short-lived cultures were characterized by a low number of chromosomally normal cells in the primary culture (42%) or by a rapid decline in chromosomally normal cells within the first 5–10 passages (72%–84% down to 32%–42% normal cells). Decreased seeding density may play a role in hastening senescence as the numerous cell divisions required for primary confluence may result in first passage cells being at a later stage of replicative potential. Other studies have shown that cultures with initially high levels of chromosomally abnormal cells (>40%) had almost 100% abnormal metaphase spreads within 9 [12] and 17 passages [26]. Since it is highly unlikely for the donor to have high levels of chromosomally abnormal cells with a range of aneuploidies, evidence of this in early passages, suggests a problem with the collection and/or culture method. Changes in chromosome content occurred in all cultures over time and the data indicate that sample collection and cell dissociation techniques have some effect on both the initial level of chromosome abnormalities and the rate of decline of chromosomally normal cells. These observations along with evidence from previous studies suggest that the chromosome content of a cell culture is directly related to its quality and longevity.

Schwartz et al. [16] had shown that chromosome instability was caused by telomere shortening. The "sticky" ends of shortened telomeres predispose the chromosomes to fusions and translocations known as telomeric associations [15], one of the major types of anomalies in chromosomally abnormal cells approaching senescence [26]. As critically short telomeres trigger the irreversible cell cycle arrest that typifies replicative senescence, Allsopp et al. [9] proposed that telomere length is a good indicator of the in vitro lifespan of cells. Our data confirm that telomere erosion occurs during serial cultivation of cells. However, a correlation between initial telomere length, its rate of decline and the type of culture established (long-lived vs. short-lived) was not clearly evident. Consequently, sample collection and cell dissociation techniques did not appear to have a major effect on telomere length. Although RTL alone was not indicative of the culture's replicative capabilities, it would appear that further information could be gained if the rate of shortening of telomere length during serial cultivation was assessed as well. This was supported by the fact that modeling chromosome stability against telomere length showed that chromosome stability was not dependent on any one telomere length factor alone but that it was highly dependent on both initial relative telomere length and its rate of change. These observations concur with previous findings where a direct relationship was noted between chromosome instability, telomere length and proliferative lifespan [16]. Assessing both chromosome and telomere dynamics of cultures would provide the necessary predictive parameters on the long-term in vitro capabilities of a cell culture.

### Effects on embryo production by SCNT

The genetic make-up of the donor cell is a crucial component for the success of novel embryo technologies such as SCNT. Embryo development is dependent upon proper reprogramming of the donor genome so that embryonic genes are appropriately expressed. A normal chromosome complement is an important prerequisite for proper gene expression to occur in the early cleavage stage embryo. It has been documented that chromosome abnormalities are detrimental to continued embryo development and are the major source of early embryonic loss [18,19]. In cattle, poor quality embryos have been shown to have higher numbers of chromosomally abnormal cells [17,20] and aneuploidy combined with increased levels of DNA fragmentation, suggestive of apoptosis, were associated with slow growing human embryos [28]. Schwartz et al. [16] had stated that chromosome instability in cells grown in vitro leads to cell cycle arrest, which triggers apoptosis, and that these chromosome abnormalities arise as a result of shortened telomere lengths. Thus, it naturally follows that shortened telomere lengths would also result in poor quality embryos. Several studies have shown that decreased telomere lengths in gametes cause a higher incidence of cytoplasmic fragmentation, which contributes to apoptosis and abnormal cleavage in early embryos [29,30]. Our data indicate that the characteristics of the donor cell cultures were directly related to the developmental potential of SCNT embryos. Since only cells obtained by collagenase digestion were used for SCNT, the effect of cell dissociation technique could not be tested. However, sample collection technique significantly affected embryo development to the blastocyst stage. Donor cells taken from passages 3 to 5 of long-lived cultures produced adequate blastocyst rates of approximately 35% and 12% for cattle and gaur, respectively. These rates are comparable to those produced by cattle and gaur SCNT in other studies [31-33], and we have demonstrated that the decreased blastocyst yield following gaur SCNT was due to the use of interspecies SCNT in which an exotic cattle donor nucleus (gaur) is directing the domestic cattle recipient cytoplasm (taurus) [31]. In this case, although the two species are closely related, sufficient differences exist between cattle and gaur at the genetic and molecular level to account for differences in embryo growth dynamics [31].

In the present study, cattle donor cells approaching replicative senescence (passage 30; 60 PDL) used for SCNT produced significantly lower blastocyst development. Similar results were reported by Lanza et al. [34] who noted only 5% blastocyst development following SCNT using bovine fibroblasts passaged to greater than 95% of their lifespan and showing morphological signs consistent with senescence. At passage 30, the cattle donor cells in this study exhibited changes in morphology indicative of the impending growth arrest, an increase in chromosome abnormalities and a decrease in telomere length. The use of gaur donor cells from short-lived cultures obtained by dart and manual punch biopsies for SCNT also produced significantly lower blastocyst development compared to the long-lived culture from the gaur ear sample. These donor cells had similar characteristics to the cattle passage 30 cells, with morphological changes and increased chromosome abnormalities. A direct correlation can be made between the alterations in cell characteristics and poor embryo development. Statistical analysis of our data indicated that blastocyst development rates of cell cultures used for SCNT were dependent on sample collection techniques and chromosome stability of the donor cell line. Studies by Slimane Bureau et al. [22] and Giraldo et al. [12] have demonstrated that the frequency of chromosome abnormalities in the donor cell line corresponds to the frequency of chromosome abnormalities in the SCNT embryos produced. Thus, a high percentage of chromosome abnormalities [12,22] or decreased telomere lengths [29,30] in the blastomeres of SCNT embryos, may result in increased levels of apoptosis or cell cycle arrest, which may be a contributing factor to their early demise.

## Conclusion

A dramatic increase in the loss of natural habitats, high rates of inbreeding, and reduced reproductive performance in both wild and domestic animals have created the necessity for genome resource banking of valuable individuals, breeds or species as a whole. Tissue samples for the establishment of cell lines are being collected, the cells grown in vitro and cryopreserved using numerous sample collection and culture establishment techniques. Although viable for use in molecular studies, these samples may prove to be inadequate for use with technologies such as SCNT. This study was performed to gain a better understanding of the effects of culture initiation techniques on culture characteristics and the subsequent developmental potential of SCNT embryos. Furthermore, we hoped to establish markers of cell viability to assist with the selection of good quality cell lines for SCNT. We have shown that sample collection and cell dissociation techniques influence the in vitro growth characteristics and lifespan, morphology, chromosome content and telomere dynamics of cell lines. The relationship between the parameters examined here and the replicative lifespan of cells in vitro, most importantly, time to primary confluence, cell morphology and chromosome content, demonstrate that various markers can be assessed in the initial phases of culture to evaluate the proliferative potential of a cell line. The significant correlation between chromosome stability and SCNT embryo development provides the researcher with a means for assessing the developmental potential of a donor cell line and thereby allowing the selection of a culture establishment protocol that produces the most genetically stable cultures. This study provides evidence that the implementation of standardized techniques for the preparation and assessment of cell lines is vital to the success of genome banking. Using proper selection criteria, cell lines with the potential for successful development can be considered for further study and banking.

## Methods

All chemicals were purchased from Sigma Chemical Co. (St. Louis, MO, USA) unless otherwise stated.

### Experimental design

#### A) Effects of tissue sample collection techniques

Samples were collected from a gaur bull as follows: a) obtaining skin using 3 mm × 10 mm biopsy darts fired from an air gun (DART), b) obtaining skin using 4 mm × 6 mm biopsy punches manually on an anaesthetized animal (PUNCH), and c) obtaining a portion of the ear post-mortem (EAR). As controls, samples were collected from a domestic bull by obtaining a portion of skin (SKIN) and ear (EAR) post-mortem.

#### B) Effects of cell dissociation techniques

Each sample collected was subjected to the following two treatments: a) explants of tissue (EXPL) and b) collagenase digestion of tissue (COLL).

### Preparation of fibroblast cell cultures

Domestic cattle (*Bos taurus*) samples from skin or ear were obtained from slaughterhouse material. Gaur (*Bos gaurus*) samples from skin or ear were obtained from gaur housed at the Toronto Zoo. Immediately upon collection, the samples were immediately placed in phosphate buffered saline (PBS) + 1% antibiotic-antimycotic (ABAM; Invitrogen Canada Inc., Burlington, ON, Canada) on ice for transport to the laboratory. Each sample was chopped into small pieces (1 mm × 1 mm) which were evenly distributed onto the bottom of a tissue culture flask for explanting or digested for 3–5 hours in 0.5% collagenase type I in Dulbecco's Modified Eagle's Medium (DMEM). All samples were cultured in DMEM + 1% ABAM + 20% fetal bovine serum (FBS) in 25 cm^2 ^tissue culture flasks at 38°C in air containing 5% CO_2 _and the medium was replaced every 3–4 days until confluence was achieved. The number of days required to achieve confluence of the primary culture was recorded for each sample (primary confluence). All the cell lines were passaged at confluency in DMEM + 1% Penicillin/Streptomycin (P/S; Invitrogen Canada Inc.) + 10% FBS at a dilution of 1:4 for approximately 2 population doublings (PDL) per passage until senescence. Senescence was characterized by the failure of the population to double after 3 weeks of culture with regular medium change. The number of PDL to senescence was recorded for each cell line. At passage 1 (P-1) and every five passages (P-5, P-10, etc) until senescence, the samples were assessed for morphology (under light microscopy using a Leica inverted microscope at 40X magnification), chromosome content and telomere length. Cells from collagenase treatments were prepared and used for SCNT at passages 3 to 5.

### Chromosome content analysis

Metaphase spreads were obtained using standard techniques. Briefly, 0.05 μg/ml of colcemid (Invitrogen Canada Inc.) was added to an actively growing culture for 2–3 hours. Cells were harvested and treated with hypotonic solution (0.075 M KCl) followed by fixation with methanol:acetic acid (3:1/v:v). The cells were spread onto slides and stained for 15 minutes with 4% Giemsa. Fifty giemsa stained metaphase chromosome spreads from each sample were examined at 100× magnification and the percent normal and abnormal cells were recorded. Rates of change of chromosome content over passages in culture (slope) were obtained from linear regression analysis of the percent normal cells.

### Relative telomere length analysis

#### a) DNA extraction

Cells harvested by trypsinization were washed once with PBS and the cell pellets were frozen at -80°C until DNA extraction could be carried out. DNA extraction and precipitation was carried out using a standard phenol-ethanol technique. Briefly, an equal volume of phenol/chloroform/isoamyl alcohol (PCI) was added to the cell pellets in a 1.5-ml microcentrifuge tube. The solution was mixed gently for 5 minutes and centrifuged for 10 minutes at 10,000 rpm at room temperature. The aqueous phase containing the DNA was removed and transferred to a new tube. This was repeated several times. An equal volume of chloroform: isoamyl alcohol (24:1/v:v) was then added and the solution was mixed gently for 2 minutes followed by centrifugation for 1 minute at 10,000 rpm. The aqueous phase containing the DNA was removed, transferred to a new tube and 2 volumes of ice-cold 100% ethanol was added. The DNA solution was mixed gently and placed at -20°C overnight or at -70°C for 1 hour. The supernatant was removed after centrifugation for 20 minutes at maximum speed. 70% ethanol was carefully added to the pellet at room temperature and centrifugation at maximum speed was repeated. The pellet was then dried at room temperature, dissolved in 20–50 μl of Tris-EDTA buffer (pH 8.0) and stored at -20°C until needed.

#### b) Relative Telomere Length (RQ-Telo Assay)

Real-time quantification of relative telomere length (RTL) for use with the LightCycler was based on the original protocol described by Cawthon [35] and modified by Gil and Coetzer [36]. Briefly, reactions were performed in triplicate in 10 μl reaction volumes (using 2 μl of sample per reaction) for control 293T DNA and all samples. The LightCycler Faststart DNA Master SYBR Green kit (Roche Diagnostics, Indianapolis, IN, USA) was used with a 3 mM final concentration of MgCl_2_. The assay included amplification of the telomere (T) and a single-copy (S) gene (BTF3) for normalization. The two reactions were prepared from a single master mix, and the telomere and single-copy gene amplifications were performed in serial. Primers for the telomere reaction were added at a final concentration of 0.2 μM and 0.3 μM for Tel 1b and Tel 2b, respectively. The BTF3 primers were added at a final concentration of 0.5 pM. The telomere primer sequences were as follows:

Tel 1b: 5'-CGGTTTGTTTGGGTTTGGGTTTGGTTTGGGTTTGGGTT-3'

Tel 2b: 5'-GGCTTGCCTTACCCTTACCCTTACCCTTACCCTTACCCT-3'.

The primer sequences for the single-copy gene were as follows:

BTF3u: 5'-AGGAACTGCTCGCAGAAAGA-3'

BTF3d: 5'-GCCCGTAATGGTGAAAGTGT-3'.

The telomere length assay consisted of a 10 minute incubation at 95°C for enzyme activation, followed by 20 cycles at 95°C for 5 seconds, 56°C for 10 seconds, and 72°C for 60 seconds and fluorescence acquisition. The single-copy gene assay consisted of a 10 minute incubation at 95°C, followed by 35 cycles at 95°C for 5 seconds, 60°C for 5 seconds, and 72°C for 20 seconds and fluorescence acquisition. Crossing points (Cp) were determined by the second derivative method using the LightCycler software.

#### c) RQ-Telo analysis

The RQ-Telo assay determines the telomere signal of a sample by normalization to the single-copy gene amplification, and is expressed relative to a control sample that is amplified in all assays. For each sample, taking the median Cp of triplicate telomere and single-copy gene amplifications minimized error. The T/S ratio for a single sample was defined as the difference in Cp between the telomere and single-copy gene, or [2^Cp(telomere)^/2^Cp(BTF3) ^] = 2^-ΔCp ^[35]. Finally, the T/S ratio of each sample was compared with the reference sample to give the relative T/S ratio by 2^-(ΔCp1-ΔCp2)^. A T/S ratio for each sample was therefore determined, and expressed as a relative T/S ratio, or relative telomere length (RTL) to the reference sample. Rates of change of RTLs over passages in culture (slope) were obtained from linear regression analysis of the RTLs.

### Somatic cell nuclear transfer

SCNT was carried out according to the protocol of Mastromonaco et al. [32]. Adult skin or ear fibroblasts were cultured as described previously and maintained in confluence for 1–3 days until processed for SCNT. Briefly, domestic cattle cumulus-oocyte complexes were collected by follicle aspiration and then cultured in modified synthetic oviductual fluid medium (mSOFaa) + 2% steer serum + hormones (E2, FSH and LH; USDA National Hormone and Peptide Program, Torrance, CA, USA) at 38.5°C in 5% CO_2 _in air for 18 hours. Oocytes with a polar body were stained with Hoechst 33342 to visualize the metaphase plate. Oocyte enucleation and donor cell transfer were carried out in modified Tyrode's medium under silicone oil. Cell fusion was carried out in 0.28 M mannitol solution + 100 μM MgCl_2 _+ 100 μM CaCl_2 _with an electrical stimulus of 1.5 kV/cm for 40 μs. Reconstructed embryos (successfully fused couplets) were activated using 5 μM ionomycin followed by 10 μg/ml cycloheximide. Embryos were cultured in mSOFaa medium + 8 mg/ml fatty acid-free BSA at 38.5°C in 5% CO_2_, 5% O_2_, 90% N_2 _for 8 days, and evaluated for blastocyst development on day 8 at 196 hours of culture.

### Statistical analysis

#### a) SCNT embryo development

For each cell line used, SCNT was repeated 3 times with N representing the total number of reconstructed embryos placed in culture. Blastocyst development rates were determined as a percentage of the number of reconstructed embryos produced. Values are reported as means ± the standard error of the mean (SEM). The effect of the treatments was analyzed using the one-way ANOVA, followed by Tukey's post-hoc analysis (Minitab for Windows, Minitab Inc., 1998), or the equivalent non-parametrical test (Kruskal-Wallis test) when samples did not meet the assumption of normal distribution or homogeneity of variance. Differences with probabilities (p) < 0.05 were considered significant.

#### b) Chromosome and telomere dynamics

To determine if there was a significant dependence of chromosome stability, defined as the chromosome content rate of change over passages in culture, on telomere dynamics, telomere values (continuous variables) including initial, final, and RTL rate of change, and source values (class variables) including animal, sample collection method, and cell dissociation method were tested by multiple linear regression analysis (SAS 8.0) against the chromosome content data. To determine if there was a significant dependence of blastocyst development on chromosome stability, blastocyst development data were tested by multiple linear regression analysis (SAS 8.0) against the chromosome content rate of change values. The regression analyses were significant with a p-value of < 0.05.

## Authors' contributions

GFM participated in the design of the study, performed the culture and SCNT experiments, including PDL, morphology, chromosome content and embryo development analyses, and drafted the manuscript. SDP performed the telomere length analyses and statistical analyses and helped to draft the manuscript. DHB contributed to the evaluation of the data and the manuscript contents. WAK coordinated the study, provided critical review of the results and manuscript contents, and acquired the funding for the research program. All authors read and approved the final manuscript.

## References

[B1] Wildt DE (1992). Genetic resource banks for conserving wildlife species: justification, examples and becoming organized on a global basis. Anim Reprod Sci.

[B2] Hayflick L, Moorhead PS (1961). The serial cultivation of human diploid cell strains. Exp Cell Res.

[B3] Pignolo RJ, Cristofalo VJ, Rotenberg MO (1993). Senescent WI-38 cells fail to express EPC-1, a gene induced in young cells upon entry into the G0 state. J Biol Chem.

[B4] Allsopp RC (1996). Models of initiation of replicative senescense by loss of telomeric DNA. Exp Gerontol.

[B5] Campisi J, Dimri GP, Nehlin JO, Testori A, Yoshimoto K (1996). Coming of age in culture. Exp Gerontol.

[B6] Balin AK, Fisher AJ, Anzelone M, Leong I, Allen RG (2002). Effects of establishing cell cultures and cell culture conditions on the proliferative lifespan of human fibroblasts isolated from different tissues and donors of different ages. Exp Cell Res.

[B7] Zhu H, Tamot B, Quinton M, Walton J, Hacker RR, Li J (2004). Influence of tissue origins and external microenvironment on porcine foetal fibroblast growth, proliferative life span and genome stability. Cell Prolif.

[B8] Hamilton ML, Van Remmen H, Drake JA, Yang H, Guo ZM, Kewitt Richardson A (2001). Does oxidative damage to DNA increase with age?. Proc Natl Acad Sci USA.

[B9] Allsopp RC, Vaziri H, Patterson C, Goldstein S, Younglai EV, Futcher AB, Greider CW, Harley CB (1992). Telomere length predicts replicative capacity of human fibroblasts. Proc Natl Acad Sci USA.

[B10] Benn PA (1976). Specific chromosome aberrations in senescent fibroblast cell lines derived from human embryos. Am J Hum Genet.

[B11] Strelchenko N (1996). Bovine pluripotent stem cells. Theriogenology.

[B12] Giraldo AM, Gomez MC, Dresser BL, Harris RF, King AL, Pope CE (2004). Chromosomal stability of African wild cat (Felis silvestris libica) somatic cells and cloned embryos [abstract]. Reprod Fert Dev.

[B13] Prakash Hande M, Samper E, Lansdorp P, Blasco MA (1999). Telomere length dynamics and chromosomal instability in cells derived from telomerase null mice. J Cell Biol.

[B14] Blackburn EH (1991). The structure and function of telomeres. Nature.

[B15] Velicescu M, Yu J, Herbert B, Shay JW, Granada E, Dubeau L (2003). Aneuploidy and telomere attrition are independent determinants of crisis in SV40-transformed epithelial cells. Cancer Res.

[B16] Schwartz JL, Jordan R, Evans HH (2001). Characteristics of chromosome instability in the human lymphoblast cell line WTK1. Cancer Genet Cytogenet.

[B17] Booth PJ, Viuff D, Tan S, Holm P, Greve T, Callesen H (2003). Numerical chromosome errors in day 7 somatic nuclear transfer bovine blastocysts. Biol Reprod.

[B18] King WA, Coppola G, Alexander B, Mastromonaco G, Perrault S, Nino-Soto MI, Pinton A, Joudrey EM, Betts DH (2006). The impact of chromosome alteration on embryo development. Theriogenology.

[B19] King WA, McFeely RA (1990). Chromosome abnormalities and pregnancy failure in domestic animals. Domestic Animal Cytogenetics.

[B20] Viuff D, Greve T, Avery B, Hyttel P, Brockhoff PB, Thomsen PB (2000). Chromosome aberrations in in vitro-produced bovine embryos at days 2–5 post-insemination. Biol Reprod.

[B21] Kawarsky SJ, Basrur PK, Stubbings RB, Hansen PJ, King WA (1996). Chromosomal abnormalities in bovine embryos and their influence on development. Biol Reprod.

[B22] Slimane Bureau W, Bordignon V, Leveillee C, Smith LC, King WA (2003). Assessment of chromosomal abnormalities in bovine nuclear transfer embryos and in their donor cells. Cloning Stem Cells.

[B23] Cristofalo VJ, Allen RG, Pignolo RJ, Martin BG, Beck JC (1998). Relationship between donor age and the replicative lifespan of human cells in culture: a reevaluation. Proc Natl Acad Sci USA.

[B24] Bartels P, Spillings B, Joubert J, van Niekerk M, Decuadro-Hansen G, Miller-Butterworth C (2003). Establishing viable cell cultures from African lions (Panthera leo) following cryopreservation of skin biopsies in CBS straws [abstract]. Theriogenology.

[B25] Saskela E, Moorhead PS (1963). Aneuploidy in the degenerative phase of serial cultivation of human cell strains. Proc Natl Acad Sci USA.

[B26] Mondello C, Riboni R, Casati A, Nardo T, Nuzzo F (1997). Chromosomal instability and telomere length variations during the life span of human fibroblast clones. Exp Cell Res.

[B27] Fauth C, O'Hare MJ, Lederer G, Jat PS, Speicher MR (2004). Order of genetic events is critical determinant of aberrations in chromosome count and structure. Genes, Chromosomes Cancer.

[B28] Findikli N, Kahraman S, Kumtepe Y, Donmez E, Benkhalifa M, Birick A, Sertyel S, Berkil H, Oncu N (2004). Assessment of DNA fragmentation and aneuploidy on poor quality human embryos. Reprod Biomed Online.

[B29] Keefe DL, Franco S, Liu L, Trimarchi J, Cao B, Weitzen S, Agarwal S, Blasco MA (2005). Telomere length predicts embryo fragmentation after in vitro fertilization in women – toward a telomere theory of reproductive aging in women. Am J Obstet Gynecol.

[B30] Liu L, Blasco M, Trimarchi J, Keefe D (2002). An essential role for functional telomeres in mouse germ cells during fertilization and early development. Dev Biol.

[B31] Mastromonaco GF (2006). Reproductive technologies for the management of an exotic bovine species in captivity. PhD thesis.

[B32] Mastromonaco GF, Semple E, Robert C, Rho J, Betts DH, King WA (2004). Different culture media requirements of IVF and nuclear transfer bovine embryos. Reprod Dom Anim.

[B33] Lanza RP, Cibelli JB, Diaz F, Moraes CT, Farin PW, Farin CE, Hammer CJ, West MD, Damiani P (2000). Cloning of an endangered species (Bos gaurus) using interspecies nuclear transfer. Cloning.

[B34] Lanza RP, Cibelli JB, Blackwell C, Cristofalo VJ, Francis MK, Baerlocher GM, Mak J, Schertzer M, Chavez EA, Sawyer N, Lansdorp PM, West MD (2000). Extension of cell life-span and telomere length in animals cloned from senescent somatic cells. Science.

[B35] Cawthon RM (2002). Telomere measurement by quantitative PCR. Nucleic Acids Res.

[B36] Gil ME, Coetzer TL (2004). Real-time quantitative PCR of telomere length. Mol Biotech.

